# Utilization of Nursing Education Progressive Web Application (NEPWA) Media in an Education and Health Promotion Course Using Gagne’s Model of Instructional Design on Nursing Students: Quantitative Research and Development Study

**DOI:** 10.2196/19780

**Published:** 2020-11-13

**Authors:** Deny Yuliawan, Doni Widyandana, Rachmadya Nur Hidayah

**Affiliations:** 1 Department of Medical and Health Professions Education Faculty of Medicine, Public Health and Nursing University Gadjah Mada Yogyakarta Indonesia

**Keywords:** mobile application, nursing students, blended learning, knowledge, satisfaction, system usability, mobile phone

## Abstract

**Background:**

Previous studies have proven that web-based learning media that offer interesting features with *the learning management system* concept could support the learning processes of nursing students. Nonetheless, it is still necessary to conduct further research on its potential as an information media that supports learning using 1 of the mobile learning methods.

**Objective:**

This study aims to develop and use the Nursing Education Progressive Web Application (NEPWA) media in an education and health promotion course for nursing students.

**Methods:**

This is a research and development study aimed at developing the NEPWA media using the Analyze, Design, Develop, Implement, and Evaluate approach and a quantitative research with descriptive and pre-experimental 1-group pretest-posttest design conducted in the Study Program of Nursing Sciences, Faculty of Health Sciences, Muhammadiyah University of Surakarta. A total of 39 nursing students in their second year of undergraduate studies participated in this study. A pretest-posttest design was used to measure any changes in the dependent variable, whereas a posttest design was used to measure any changes in the independent variables.

**Results:**

After using the NEPWA media, there was a significant increase in the student knowledge variable (N=39; knowledge: *P*<.001; 95% CI 23.88-33.14). In terms of student satisfaction with the learning process using Gagne’s model of instructional design, most of the students were satisfied, with a mean score of ≥3. In addition, the results of the measurement using the System Usability Scale on the NEPWA media showed that NEPWA has good usability and it is acceptable by users, with a mean score of 72.24 (SD 8.54).

**Conclusions:**

The NEPWA media can be accepted by users and has good usability, and this media is designed to enhance student knowledge.

## Introduction

Learning in the age of digital transformation has different characteristics compared with that in the past decades. The learning model in the digital age has created a new trend that uses electronic media known as mobile learning (m-learning), which offers many benefits such as flexible and portable learning designed without space-time constraints [1].

In general, the majority of internet users are middle and high school or university students aged 10-24 (18.40%) years and 25-34 (24.40%) years [2], respectively, whereas in the context of health sciences, the ages of 10-18 years are a period of preparation before individuals enter higher education, where, in this age group, the majority of internet users are at the high school education level. One of the unique features of web-based learning is the availability of an independent, effective, and efficient learning environment where users can repeatedly access information and skills to practice without space-time constraints [3], which could benefit the learning in both campuses and clinics [4].

Students are interested in using web-based learning through mobile apps as they offer many interesting features [5-8]: mobile apps can be installed in every Android smartphone [9] and are flexible to use. These are the factors that attract users [10]. Learning media using m-learning, which can be applied in lectures using demonstration learning methods to become more interactive, are popular and commonly used in the health sector [1]. On the other hand, 1 of the limitations of traditional lecture methods is that they can be boring as lecturers deliver materials only via texts, making the lectures passive. Thus, it is necessary to address this issue by using effective learning media, one of which is the use of web-based learning media in the form of mobile apps with a simple concept of a *learning management system* that could stimulate interactions during lectures and provide feedback to students; therefore, it is necessary to design learning strategies well [1].

This is in line with some previous studies, which showed that app media that integrate web-based lectures using electronic learning (e-learning) media can increase student knowledge about the given courses [11-13]. On the basis of a preliminary study by interviewing one of the lecturers and students at the Study Program of Nursing Science, the Faculty of Health Sciences, Muhammadiyah University of Surakarta, it can be concluded that some of the learning media used by lecturers are in the form of PowerPoint slides, handouts, and videos from YouTube, as well as the use of one of the principles of Gagne’s instructional design, that is, pretest. Thus, it is necessary to innovate web-based learning media to be used during lectures to assist students in studying.

The interactive learning strategies in this lecture were designed using the 9-step Gagne’s model of instructional design with a teaching method using blended learning. Gagne's model of instructional design includes instructions that allow learning processes to be effective [14,15].

On the basis of the abovementioned points, the problems of this study were formulated as follows: “Do the development and utilization of the NEPWA media on education and health promotion course influence the improvement of nursing students’ knowledge?”

The objective of the study was to develop and utilize the Nursing Education Progressive Web Application (NEPWA) media in an education and health promotion course among nursing students.

## Methods

**Figure 1:**
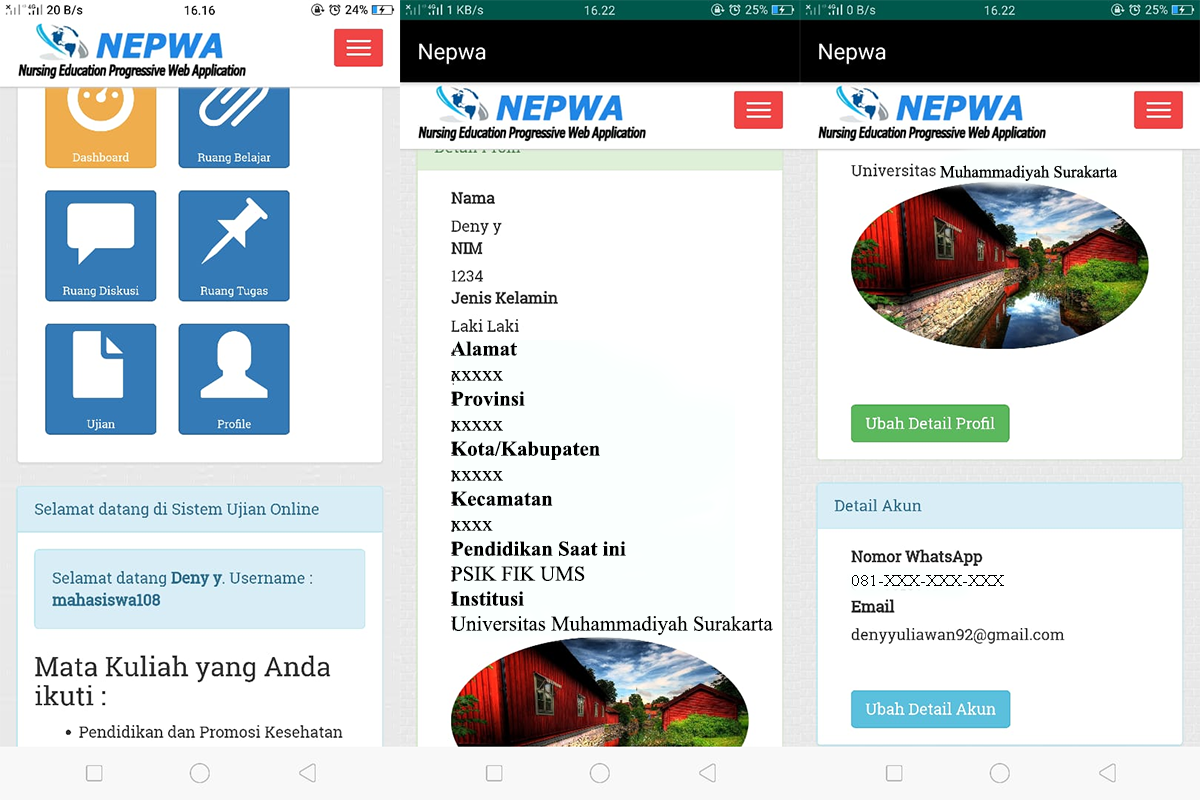
Display of the student profile page on the Nursing Education Progressive Web Application media.

This was a research and development study [16]. The development of the NEPWA media used the Analyze, Design, Develop, Implement, and Evaluate approach [17]. The first needs analysis was conducted according to the needs of the researchers to develop apps and websites in the form of both software and hardware as well as materials in the NEPWA media. Afterward, the NEPWA media were designed in accordance with the needs of lectures by adjusting to Gagne’s model of instructional design*, *including the features of learning material rooms, discussion rooms, assignment rooms, and test rooms. The design for NEPWA media was then developed and made by a team expert app and web programmer based on the researchers’ designs. The web-based learning media created was then named NEPWA. The NEPWA media was developed into 3 sections with their own features, which were correlated with one another, available in the form of both websites and apps. The first result of the NEPWA media was student page, which consists classrooms, discussion rooms, assignment rooms, and test rooms and user profile features, as shown in Figure 1.

**Figure 2:**
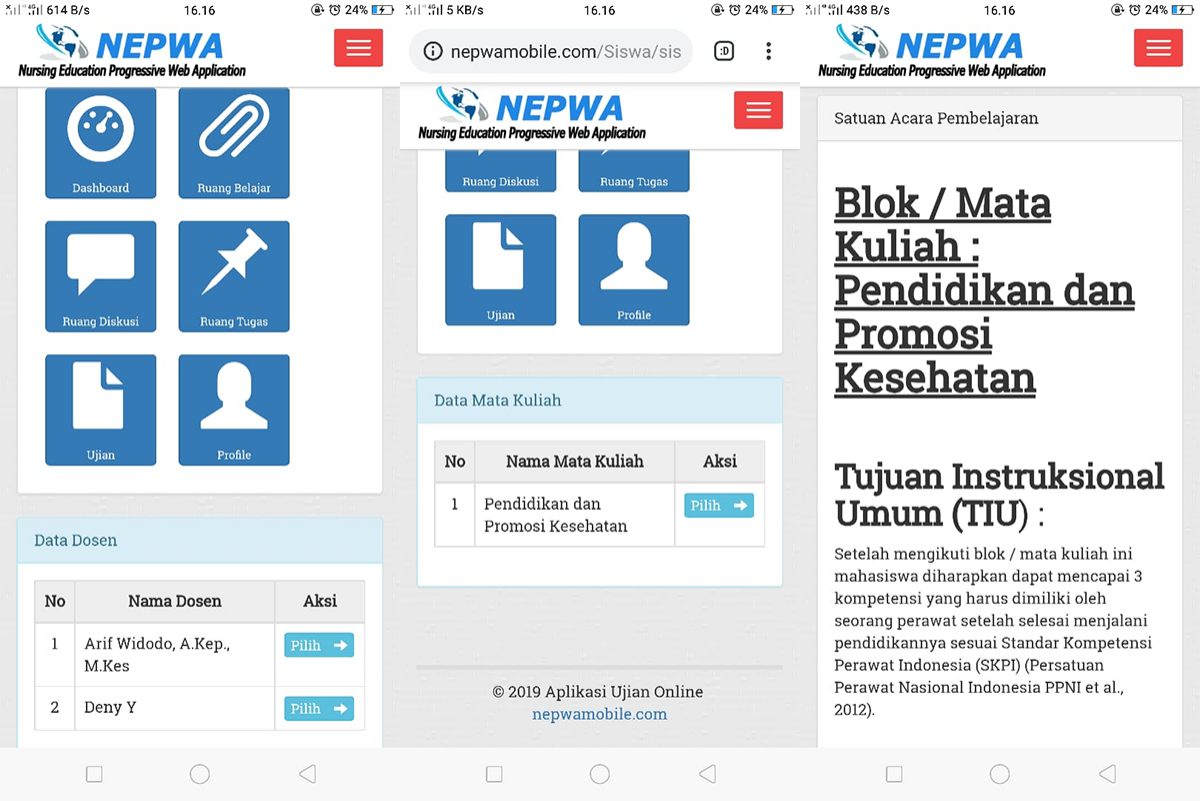
Display of the student classroom page on the Nursing Education Progressive Web Application media.

Next, students can use the classroom feature that contains instructions on how to use the NEPWA media, modules, course units, and learning materials in the forms of PowerPoint slides, infographics, and the resume of learning materials, as shown in Figure 2.

**Figure 3:**
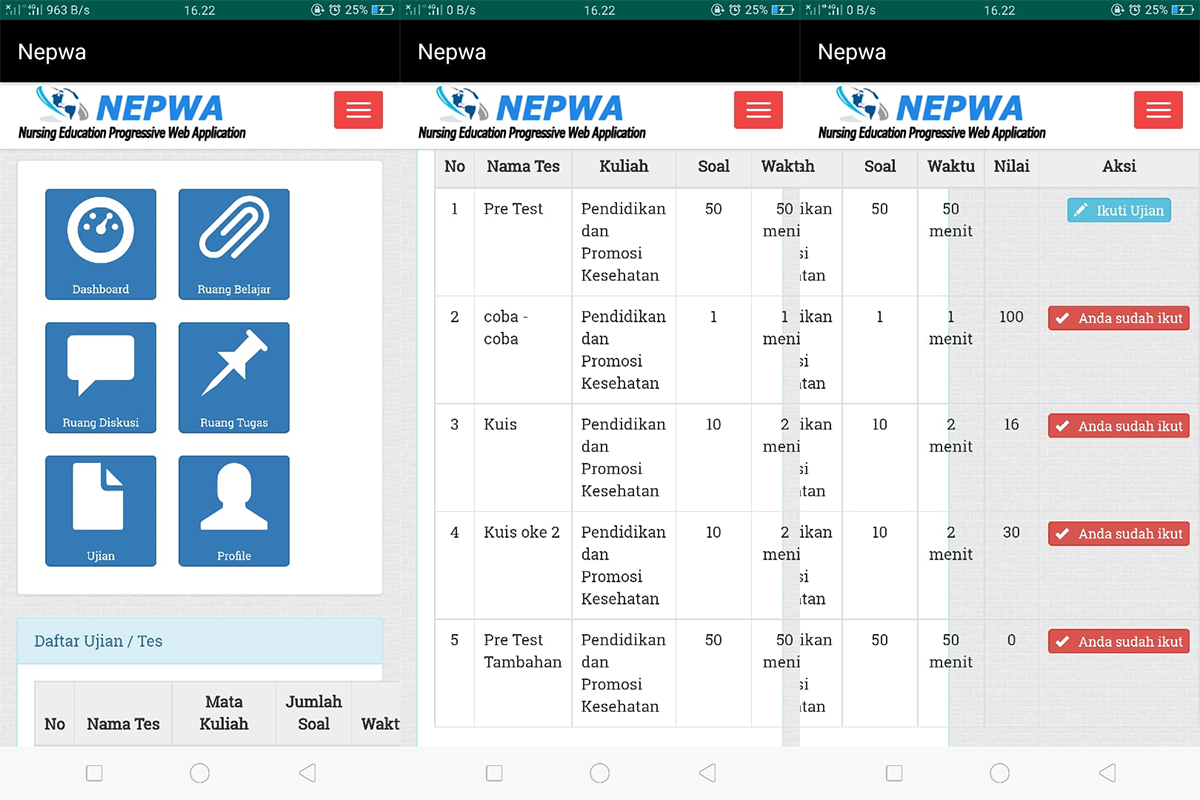
Display of the test room page on the Nursing Education Progressive Web Application media.

In addition, students’ test room features are also available, as shown in Figure 3.

The strengths of the test room in the NEPWA media are, first, the questions that simultaneously show up on the account of 1 student are not the same as those showing up on the accounts of other students; thus, students could focus on the questions showing up on their own page. Second, the duration and number of questions set by lecturers are also displayed. Third, the scores for each of the tests taken by students will show up at the end of the test session as taken by each student.

**Figure 4:**
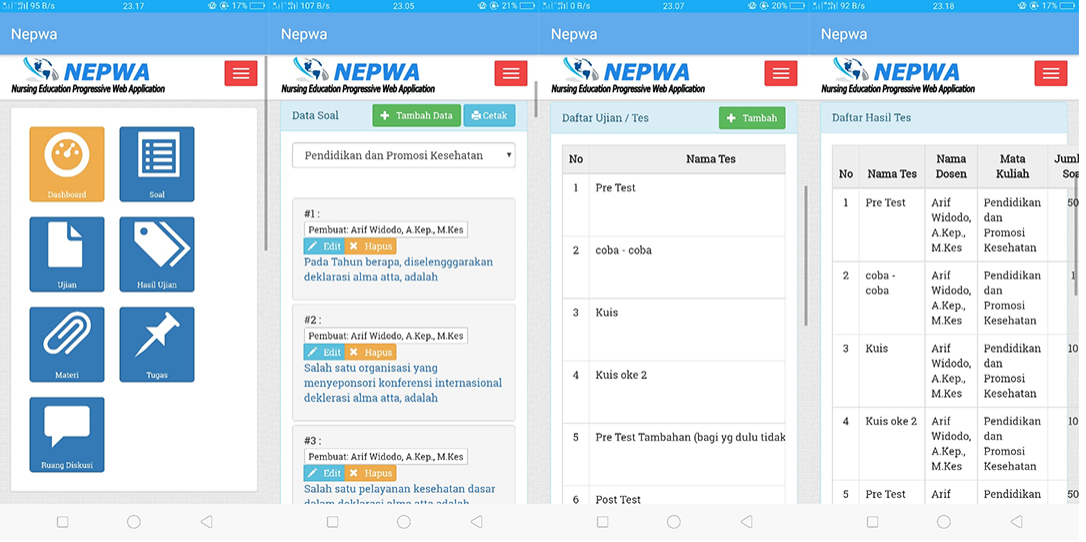
Display of the lecturer page containing the question bank and list of tests on the Nursing Education Progressive Web Application media.

For the second results, the development of the lecturer page display on the NEPWA media contains the features of the question room, test room, test score, learning material room, assignment room, and discussion room, as shown in Figure 4.

The benefits are question rooms for each course and examination registration room for students that can be managed directly by the lecturers are available, such as setting the name of the examination, the number of questions tested, and the examination duration. In addition, there are also learning outcome rooms that display the scores of all students who take each examination.

**Figure 5:**
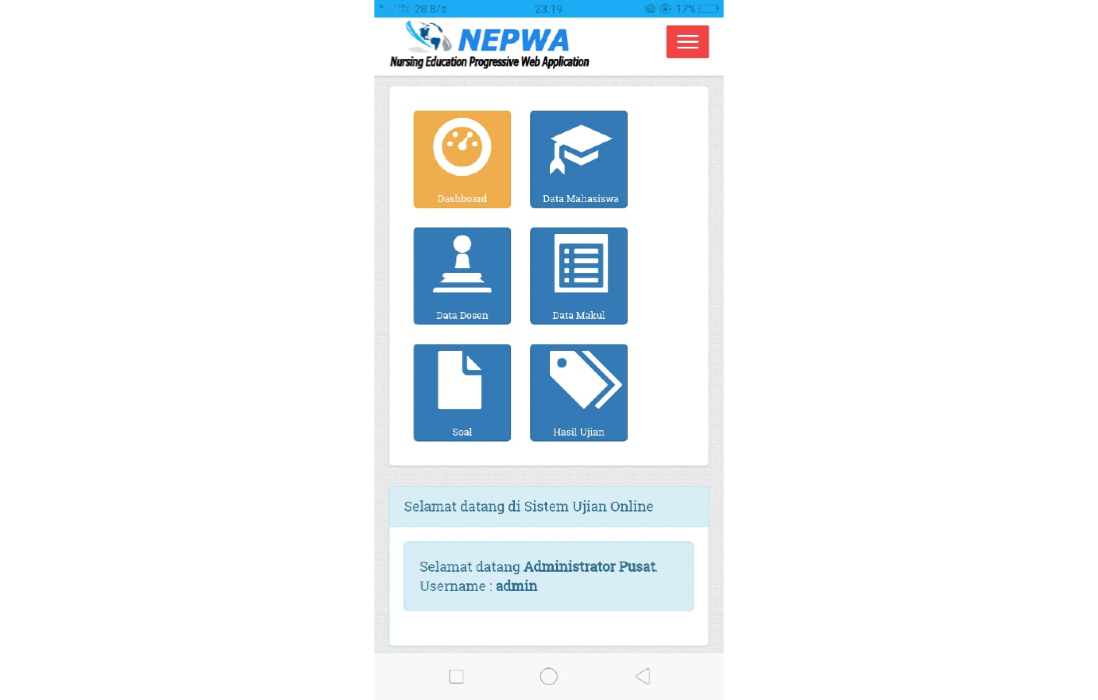
Display of the administrator page containing student data on the Nursing Education Progressive Web Application media.

For the third result, the development of the admin page on the NEPWA media consisted of the features of student data, lecturer data, course description data, questions, and the test results, as shown in Figure 5.

**Figure 6:**
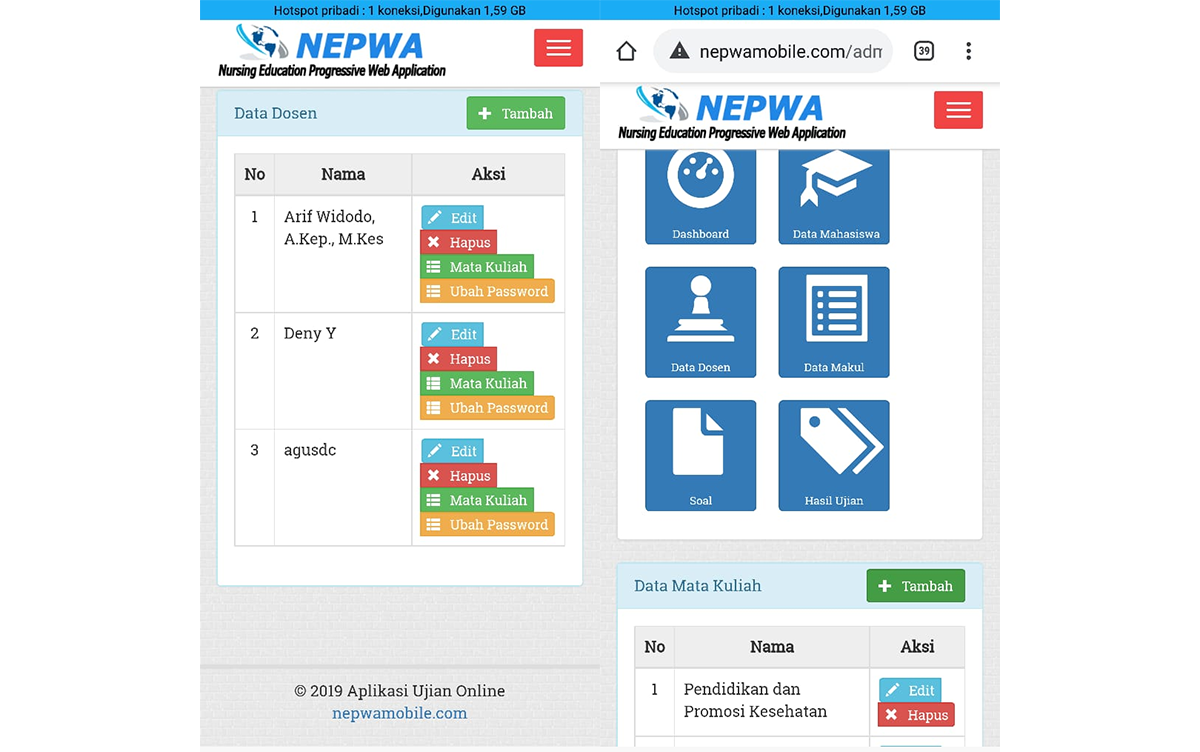
Display of the admin page containing data on lecturers and courses on the Nursing Education Progressive Web Application media.

The admin page displays student data that can only be managed directly by 1 administrator. The data include student name and number, study program*,* and *automatic username-password* to be used by students to log in to their accounts on the NEPWA media. In addition, there are also features of lecturer data and course description data, as seen in Figure 6.

The features of lecturer data and course description data that can only be managed directly by 1 administrator are available. The data include the *lecturer name and automatic username-password* to be used by lecturers to log in to their account on the NEPWA media; these are linked to the features of course description. Moreover, the feature of course description data can be inputted by the admin by entering the name of the course that has been approved by the study program.

The next step is the implementation of the NEPWA media that had been created. The NEPWA media was tested and rated by the students. Once the respondents tried using the NEPWA media, they were then asked to provide feedback by filling out a questionnaire to measure the System Usability Scale (SUS) of the NEPWA media, which was conducted after the posttest was completed.

**Figure 7:**
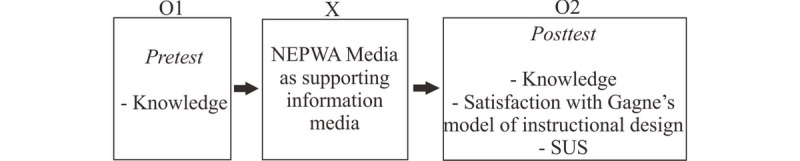
Scheme 1 Quantitative study with descriptive and pre-experimental 1-group pretest-posttest design.

The next step was when the NEPWA media was already tested and ready to use. Subsequently, research using the NEPWA media was conducted. This was a quantitative study with descriptive and pre-experimental one-group pretest-posttest designs [16,18,19], presented in Figure 7. The group descriptions in Figure 7 are as follows:

- O1: Pretest in the sample group
- X: Treatment (NEPWA media)
- O2: Posttest in the sample group

The following 3 aspects were measured:

1. Student learning outcomes in the factual knowledge (cognitive) domain were measured using multiple-choice questions before and after the face-to-face session as the pretest and posttest in the sample group.
2. Satisfaction with Gagne’s model of instructional design was measured after students completed the face-to-face and posttest sessions. The satisfaction questionnaire was adopted from the study by Buzzetto More [20] and used in a previous study by Fandianta [13] in face-to-face and web-based sessions (Multimedia Appendix 1).
3. SUS was measured after students completed the face-to-face and posttest sessions using a reliable and validated questionnaire by Sharfina and Santoso [21] and Mohamad Marzuki et al [22] (Multimedia Appendix 2).

In this study, researchers used a sample of undergraduate nursing students (N=39) calculated using the Harry King’s nomogram with saturation [16,23] in the fourth semester of the second year at the Muhammadiyah University of Surakarta between June 2019 and July 2019. These students were asked to participate in this study, and no one refused. No student was excluded, as all of them met the important inclusion criteria of the study, that is, being registered as active students in the education and health promotion course and having a smart phone or tablet to access the NEPWA media. The study had 1 variable, namely, the independent variable that measured satisfaction with Gagne’s model of instructional design through the NEPWA media using a satisfaction questionnaire [20] in the Indonesian version [13] adjusted to Gagne’s model of instructional design [14,15], and the usability of the NEPWA media was measured using the SUS score [24] in the Indonesian version [21] and the Malay language version [22]. The dependent variable then measured student knowledge using 50 multiple-choice questions with cognitive skill level C2 according to Bloom’s taxonomy. Meanwhile, the data collection technique used printed paper for the SUS questionnaire; the NEPWA media was used for the results of the pretest-posttest*.* This study consisted of 3 stages.

**Figure 8:**
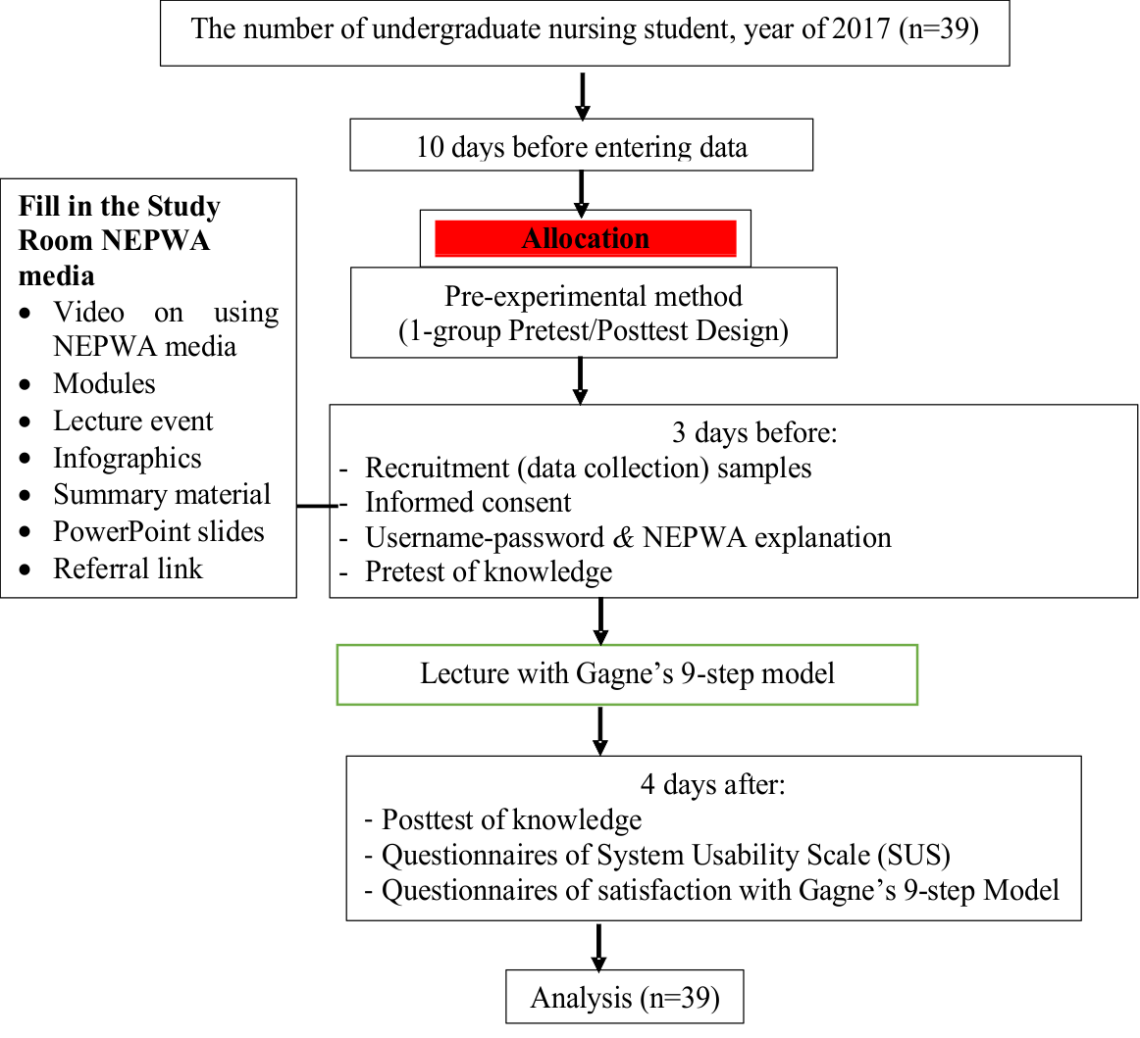
Scheme 2 Research Implementation.

The preparation stage lasted 4.5 months, where the researchers had coordinated with lecturers during the process of designing the module as well as pretest-posttest questions to test student knowledge. In addition to these activities, the researchers also coordinated with the programming team during the process of designing the NEPWA media. Once completed, the researchers pilot tested the NEPWA media, which contained a module to the supervisors, some students of Master of Medical and Health Profession Education and students of Magister of Nursing programs who were in their final semester. Finally, the implementation stage lasted 2 weeks, as shown in Figure 8.

Once all the data were collected and entered into Microsoft Excel, analysis of the characteristics of the research subjects and interpretation of the NEPWA media system usability using SUS score [25,26] were conducted. Mean SUS score ≥68 is categorized as having good usability [21,22,25]. The next step was to conduct an analysis of satisfaction with Gagne’s model of instructional design. The difference between the pretest-posttest scores in terms of the students’ factual knowledge was then determined by analyzing the data of score difference that had normal distribution. Thus, differences could be tested using a parametric statistical test, that is, the paired sample *t* test*.*

In conducting this study, the researchers obtained research ethics approval from the Committee of Research, Medical and Health Ethics, Faculty of Medicine, Public Health and Nursing at Gadjah Mada University in Yogyakarta with Ref: KE/FK/0290/EC/2018, dated March 18, 2019.

## Results

### NEPWA Media

The learning media developed by the researchers and a team of programmers ran well. The web-based learning media, called NEPWA, is accessible through the website [27]. It can also be installed as an Android app.

### Characteristics of the Subjects

The research could be implemented according to the research design. The students participated in this study voluntarily after they were given an explanation about the research objectives and processes. The selection of the students as the research participants took place right before the pretest was completed after a lecture session on education and health promotion ended, starting on June 17, 2019, in 1 class. There were 39 students in 1 classroom, and 100% (39/39) of them agreed to be the research subjects, and they all met the inclusion criteria. Consequently, these 39 students fully participated in the research activities, and the results were further analyzed.

The characteristics of the research subjects were in the form of demographic data, including age, sex, and grade point average. The distribution normality of the participants’ characteristic data was analyzed using the Shapiro-Wilk criteria: data are said to be normally distributed if the *P*>.05. The detailed characteristics of the respondents are shown in Table 1.

**Table 1:** Characteristics of the respondents (N=39).

Characteristics^a^		Respondents
Age (years), median (min-max)		20 (19-21)
Grade point average, mean (SD)		3.54 (0.17)
**Sex, n (%)**		
	Male	6 (15)
	Female	33 (85)
**Frequency of using web-based learning media during lectures, n (%)**		
	Not using at all	1 (3)
	<30 min	8 (21)
	30-60 min	21 (54)
	1-2 hours	8 (21)
	3-4 hours	1 (3)

^a^Data collection process was carried out in 2019 using the first or primary source.

### Student Satisfaction With the Use of NEPWA Media

The survey of satisfaction with the NEPWA media filled out by student participants included media used to access the internet (mobile phones, laptops, tablets, and personal computers), internet access (Wi-Fi and cellular data), and the frequency of using instructional media. The descriptive analysis results indicated a good level of satisfaction with the use of the NEPWA media. In fact, most of the students used mobile phones as hardware and cellular data to access information provided on the NEPWA media. Besides, the majority of them (>40%) accessed the NEPWA media 1-2 times per day for <30 min before, during, and after the education and health promotion course took place. In addition, the most commonly used and the most useful features were the learning material room and the test room, with a frequency >64%. Meanwhile, the brief answers on the satisfaction questionnaire regarding the use of the NEPWA media resulted in some written statements from the participants, with 3 questions that covered several subthemes of the resume, as follows:

*Good aspects of NEPWA media*

- Learning has become more effective, efficient, time-saving, and simpler because it can be done anytime and anywhere.
- The learning materials can be read repeatedly, making it easier for students to study.
- It is easier to use all available features.

*Some aspects of NEPWA media that need to be improved*

- It is necessary to perform system monitoring, preventing it from being disrupted easily.
- The display should be made more attractive, especially the display of user profile, learning material room, and discussion room.

*Satisfaction with the use of NEPWA media*

- Availability of many test practice questions.
- Availability of comprehensive learning materials.

### SUS of NEPWA Media

On the basis of the results of the distribution analysis, the NEPWA media had good usability and could be accepted by the users in this study, that is, the measurement uses the system usability variable of the NEPWA media of all students (N=39). The mean SUS score per statement can be seen in Table 2.

**Table 2:** Mean System Usability Scale score for each statement.

Number	Statement items^a^	Values, mean (SD)
1	I think that I would like to use this system	3.03 (0.28)
2	I found the system unnecessarily complex	2.95 (0.51)
3	I thought the system was easy to use	3.05 (0.39)
4	I think that I would need the support of a technical person to be able to use this system	2.56 (0.91)
5	I found that the various functions of the system were well integrated	3.15 (0.43)
6	I thought there was too much inconsistency in this system	2.87 (0.52)
7	I would imagine that most people would learn to use this system quickly	3.05 (0.61)
8	I found the system very cumbersome to use	3.03 (0.49)
9	I felt very confident using the system	3.03 (0.67)
10	I needed to learn a lot of things before I could get going with this system	2.18 (1.02)

^a^Data collection process was carried out in 2019 using the first or primary source.

**Figure 9:**
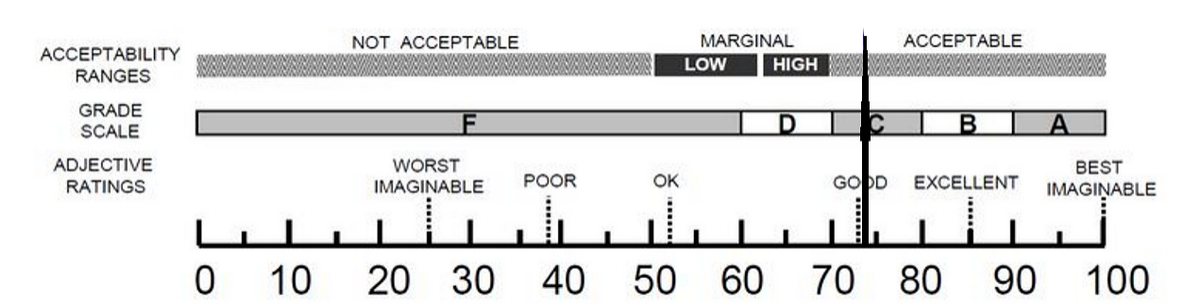
Results of System Usability Scale score on the Nursing Education Progressive Web Application media. SUS: System Usability Scale.

Table 2 presents the mean score for each of the statements of all the subjects, and it can be seen that the positive statements 5, 3, 7, 1, and 9 had a mean score of 3 (ranging from 0 “strongly disagree” to 4 “strongly agree”), which tended to be higher than the negative statements. Next, the total score of each respondent was multiplied by 2.5, resulting in the SUS score of each respondent. The total SUS score was obtained by summing the SUS scores of all the respondents, resulting in a total score of 2817. Afterward, the mean SUS score was obtained by dividing the total SUS score by the number of respondents, resulting in a score of 72.24 (SD 8.54), which indicated that the NEPWA media has good usability, meaning that it is below the acceptable range for the user, as shown in Figure 9.

### Satisfaction Scale With Gagne’s Model of Instructional Design

On the basis of the results of the satisfaction analysis, the majority of the respondents in this study were satisfied with the use of Gagne’s model of instructional design in the course using the NEPWA media from all the students (N=39). The mean score of satisfaction with Gagne's model of instructional design using the NEPWA media per statement item is presented in Table 3.

**Table 3:** Mean score of satisfaction with Gagne’s model of instructional design using the Nursing Education Progressive Web Application media for each statement.

Number	Statement item^a^	Values, mean (SD)
1	Overall, I am satisfied with learning that uses this teaching media	2.92 (0.58)
2	I am satisfied with learning that uses this teaching media as a source of information	2.97 (0.54)
3	I am satisfied with the contents available in this learning media	2.95 (0.51)
4	I always use this learning media to answer questions	2.62 (0.71)
5	I am satisfied with the contents or materials available in this learning media to assist me in learning	3.15 (0.63)
6	I believe that this learning media could enhance my understanding	3.00 (0.51)
7	I am satisfied with the brief summary available in the PowerPoint slides in this learning media	3.15 (0.49)
8	I feel that this learning media provides instructions/keywords available	2.79 (0.73)
9	I am satisfied that I can have discussions using this learning media	3.00 (0.51)
10	I feel that the web-based discussion room available in this learning media could help me gain understanding of learning materials	2.97 (0.43)
11	I am satisfied that I can interact with lecturers and other students through the web-based discussion rooms available in this learning media	2.92 (0.62)
12	I am satisfied that I could take tests using this learning media	3.08 (0.42)
13	I feel that the web-based tests available in this learning media are easier to use	3.03 (0.54)
14	I am satisfied when I receive scores immediately after taking tests using this learning media	3.03 (0.43)
15	I am satisfied that I can send assignments on the web using this learning media	3.00 (0.46)

^a^Data collection process was carried out in 2019 using the first or primary source.

On the basis of the results from Table 3, it can be seen that the mean score of the statement items was ≥3, indicating that the students were satisfied with several statement items or features available in the NEPWA media using Gagne’s model of instructional design, including:

1. Satisfaction with the modules or materials available in the learning media.
2. Satisfaction with the brief summary on the PowerPoint slides available in the learning media.
3. Satisfaction because they could take tests using the learning media.
4. Satisfaction because they could easily take web-based tests using the learning media.
5. Satisfaction because they received a score immediately after they completed a test using the learning media.
6. Satisfaction because they could send assignments on the web through the learning media.
7. Satisfaction because they could have discussions using the learning media.

### Knowledge-Level Distribution Analysis on the NEPWA Media

The results from the analysis showed that the data were normally distributed. There was an increase in the variable of knowledge level using the NEPWA media among all the students (N=39). The increase in the mean score before and after using the NEPWA media is presented in Table 4.

**Table 4:** Results of analysis of knowledge levels on the Nursing Education Progressive Web Application media.

Results^a^	Pretest, mean (SD)	95% CI	Posttest, mean (SD)	95% CI
Knowledge levels	44.41 (12.36)	40.40-48.42	72.92 (14.62)	68.18-77.66

^a^Data collection process was carried out in 2019 using the first or primary source.

On the basis of the results in Table 4, it can be seen that the pretest mean score was 44.41 (SD 12.36), which increased to 72.92 (SD 14.62) in the posttest after using the NEPWA media. This indicates that there was an increase in the mean score where the posttest score was higher than the pretest score.

### Analysis of Differences in Student Knowledge Improvement Using the NEPWA Media

The level of student knowledge before and after using the NEPWA media and the difference between these two were found to have a normal data distribution; thus, the analysis used a paired sample *t* test to determine the significance of the changes in the level of student knowledge before and after using the NEPWA media. From the test results, the mean score of student knowledge before using the NEPWA media was 44.41 (SD 12.36), which significantly increased (*P*<.001) by 23.51 (SD 14.29) to 72.92 (SD 14.62) after using the NEPWA media. The detailed results of the paired sample *t* test on the level of student knowledge are presented in Table 5.

**Table 5:** Differences in the levels of student knowledge before and after using the Nursing Education Progressive Web Application media analyzed through paired *t* test.

Levels of student knowledge^a^	N	Mean (SD)	Difference in mean (SD)	95% CI	*P* value
Before using the NEPWA^b^ media	39	44.41 (12.36)	28.51 (14.29)	23.88-33.14	<.001
After using the NEPWA media	39	72.92 (14.62)	N/A^c^	N/A	N/A

^a^Data collection process was carried out in 2019 using the first or primary source.

^b^NEPWA: Nursing Education Progressive Web Application.

^c^N/A: not applicable.

Table 5 shows that the *P* value**(<.001) is less than the α value (.05); thus, it can be concluded that there were significant changes in the score of knowledge of education and health promotion before and after using the NEPWA media. These results were also supported by the pretest mean score of knowledge of 44.41, which increased to 72.92 in the posttest after using the NEPWA media, and there was a significant increase in the score after using NEPWA media (0.449)*.* In conclusion, there is a significant change in the score of knowledge before and after using the NEPWA media. This indicates that the use of the NEPWA media affects students’ knowledge. In other words, the NEPWA media can increase knowledge.

## Discussion

### Summary

The variables of knowledge, satisfaction with the learning processes, and system usability in the NEPWA media showed an increase in differences and acceptance by users. The NEPWA media among student users can be said to have good system usability with a range of acceptance to be acceptable by the users. Next, in terms of the variable of satisfaction with Gagne’s model of instructional design, the majority of students were satisfied. The variable of student knowledge then increased significantly compared with the conditions before using the NEPWA media.

Students’ prior knowledge is an adequate key so that students’ interest in the learning process can be achieved properly. This should be supported by adequate learning media, for example, the use of web-based learning media in the form of NEPWA media as supporting information media to improve cognitive learning outcomes. Learning media in the form of e-learning is an innovative app to support the teaching and learning activities of both lecturers and students [28], of which the interactive functions shall be improved to accommodate the needs of students [29] as well as the development of the NEPWA media using Gagne’s model of instructional design to support active learning and provide flexibility in improving the quality of student classroom learning by creating creative media content [30].

To allow for the materials to be well delivered to students, a blended learning method between web-based learning methods using app or web media combined with appropriate teaching methods (face-to-face) was used, where blended learning can be used to facilitate communication and improve student learning outcomes [29]. In this case, web-based media use NEPWA media as supporting information media. In line with an earlier study [31], students can feel the benefits of implementing web-based interventions in learning, but they are not yet fully prepared to leave the traditional learning methods, including face-to-face lectures. Consistent with some previous studies [32,33], electronic-based learning provides flexible teaching methods, but both electronic-based learning and traditional teaching methods should be used simultaneously to create a superior learning style, especially for nursing courses. The explanation of the variables in this study is as follows:

#### Increase in Student Knowledge

Many previous studies have shown that web-based learning media using the blended learning approach in health profession education, in general, could significantly improve learning outcomes in terms of students’ cognitive level compared with traditional learning methods. This is based on the fact that web-based learning can optimize learning media by combining images, texts, and video, among others. The media displayed on the NEPWA media are in the form of PowerPoint slides, learning material resumes, interactive quizzes, and additional infographics, which cannot be displayed on print media. Infographics are visualization tools that can be used to improve understanding and attractiveness [34].

This is consistent with the Cognitive Theory of Multimedia Learning [35-38], where multimedia displays can be in the form of text, images, and sound. In line with this theory, there were similar findings in another study [34] where students felt that the summary infographics provided by lecturers are very interesting and useful in understanding the topics being taught. This is in accordance with the Cognitive Load Theory [39-41], which states that humans have limited memory capacity; thus, the materials being learned should be summarized according to the topic of learning. The results of a previous study [42] showed that there are no statistical differences between the groups of students learning via a web-based app or learning in face-to-face sessions in terms of knowledge, although the web-based app media is superior to face to face; because web-based app media is able to optimize learning media with a combination of pictures, writing, and videos, [43] and the effects of learning methods on learning outcomes are very situational [44].

#### Satisfaction With Gagne’s Model of Instructional Design in Learning Media

The use of Gagne's model of instructional design in the learning process using the NEPWA media was well implemented and obtained satisfactory scores on several statement items. This is based on the fact that the first highest mean score was achieved by statement 5, “I am satisfied with the contents of the modules or materials available in this teaching media to help me study”; the second highest mean score was achieved by statement 7, “I am satisfied with brief summaries on PowerPoint slides in this instructional media”; and the third highest mean score was achieved by statement 12, “I am satisfied that I can take examination using this instructional media.” Therefore, it is expected that the high level of satisfaction with Gagne’s model of instructional design in the NEPWA media can support student learning activities and help them achieve maximum competencies.

Many previous studies have also shown that users of web-based learning media using a blended learning approach in health professions education, in general, are satisfied with the teaching methods. In line with another study [32], when students have inadequate skills to use web-based learning media, it influences how they communicate effectively. However, the groups of students who used web-based learning media were satisfied with the program as a teaching method. The findings of another study [45] showed that positive results from web-based teaching and learning practices can increase student satisfaction and retention (the ability to store and memorize learning materials). Nonetheless, there are many challenges in performing the abovementioned activities, namely, the need to improve infrastructure and institutional support (training for educators to enhance their information technology competencies and support for multidisciplinary teams to improve web-based learning design) [45].

#### SUS

The NEPWA media has been well implemented. The results of the SUS were based on the highest mean score in statement 5, “I feel that the features of this system are working properly,” and the next highest mean score was in statement 3, “I feel that this system is easy to use.” In this way, it is expected that this innovative NEPWA media can support the teaching and learning activities of both lecturers and students and have a significant positive effect to continue and enjoy its uses because its features are working properly. However, the sustainable use of the NEPWA media is strongly influenced by the positive and subjective experiences of students, such as being confident and happy when learning by using the NEPWA media to increase learning productivity [28].

The result of the SUS score developed in the NEPWA media was 72.24, indicating that the NEPWA media has good usability with scale C, with a range of acceptance that is acceptable to users [22,25]. This is in line with some previous studies [21,22,25], where systems or products with SUS scores >68 are considered to have a good usability category; thus, the NEPWA media has good usability. As mentioned in another study [46], the SUS score of the Moodle-based m-learning management system named Student Centered e-Learning Environment was 71.25; hence, this app can be categorized as having good usability. The fact that the NEPWA media obtained a good SUS score proved that this media is very easy to use and acceptable by student users. In fact, some of the factors that influence the SUS score are user experience, in which user groups who have repeatedly used it previously tend to give a higher SUS score compared with the group of new users [47]. Next, easy installation is crucial as users are more satisfied when an app is easily installed on a smart phone without having to download first [48].

The study had several limitations. For example, there were no features for the statistical calculation of scores based on answers to questions, the learning materials, and questions on the NEPWA media had not been validated, and there was no repeated measurement of knowledge to determine its impact on learning outcomes.

### Conclusions

Web-based learning media called the NEPWA media is designed to increase student knowledge. This research showed that the NEPWA media is acceptable to users, useful, has good usability, and offers flexible learning for students.

With the potential to increase knowledge and have a good usability score, the NEPWA media can be used as supporting information media when implementing blended learning combined with Gagne’s model of instructional design with a good level of satisfaction with the learning processes. In other words, the NEPWA media can be used as alternative learning media, in addition to print media, which can help learning processes.

### Recommendations

Future researchers are expected to conduct a validity test and add qualitative research data to support quantitative findings, which can be used to examine repetition of learning outcomes and knowledge retention. In the future, the development of the NEPWA media should focus on adding statistical calculation features to determine the number of answers per multiple-choice question examination results from users and enhancing the interactive skills of the features to be used in various fields, particularly nursing.

## Appendix

Multimedia Appendix 1 Questionnaires on satisfaction with Gagne’s Model.

Multimedia Appendix 2 Questionnaires related to the System Usability Scale.

## Abbreviations

e-learning: electronic learning
m-learning: mobile learning
NEPWA: Nursing Progressive Web Application
SUS: System Usability Scale
