# Supplementary material for: Utilization of Nursing Education Progressive Web Application (NEPWA) Media in an Education and Health Promotion Course Using Gagne’s Model of Instructional Design on Nursing Students: Quantitative Research and Development Study
Source: JMIR Nurs. 2020 Nov 13;3(1):e19780. doi: 10.2196/19780 (PMC8279452; doi:10.2196/19780)
Supplement: Multimedia Appendix 1 [file nursing_v3i1e19780_app1.docx]

Tabel 3 Rerata skor kepuasan desain instruksional Gagne menggunakan media NEPWA per butir pernyataan

| No | **Item Pernyataan** | Rerata |
| --- | --- | --- |
| 1 | Secara keseluruhan, saya puas dengan pembelajaran yang menggunakan media ajar ini | 2,92 |
| 2 | Saya puas dengan pembelajaran yang menggunakan media ajar ini sebagai sumber informasi | 2,97 |
| 3 | Saya puas dengan konten yang tersedia di media ajar ini | 2,95 |
| 4 | Saya selalu menggunakan media ajar ini untuk menjawab pertanyaan | 2,62 |
| 5 | Saya merasa puas dengan isi modul/ materi yang tersedia di media ajar ini, untuk membantu saya belajar | **3,15** |
| 6 | Saya yakin media ajar ini meningkatkan pemahaman saya | **3,00** |
| 7 | Saya puas dengan catatan kuliah/ ringkasan singkat pada powerpoint di media ajar ini ini | **3,15** |
| 8 | Saya merasa media ajar ini memberikan petunjuk/ kata kunci yang di sediakan | 2,79 |
| 9 | Saya merasa puas dapat berdiskusi pada media ajar ini | **3,00** |
| 10 | Saya merasa ruang diskusi online di media ajar ini, membantu saya memahami materi kuliah ini | 2,97 |
| 11 | Saya merasa puas dapat berinteraksi dengan dosen dan mahasiswa lain di ruang diskusi online menggunakan media ajar ini | 2,92 |
| 12 | Saya merasa puas dapat mengikuti ujian menggunakan media ajar ini | **3,08** |
| 13 | Saya merasa ujian online pada media ajar ini lebih mudah digunakan | **3,03** |
| 14 | Saya puas ketika saya mendapatkan nilai langsung setelah mengikuti ujian menggunakan media ajar ini | **3,03** |
| 15 | Saya merasa puas dapat menuliskan tugas secara online melalui media ajar ini | **3,00** |

Sumber: Data Primer, 2019
