# Supplementary material for: Utilization of Nursing Education Progressive Web Application (NEPWA) Media in an Education and Health Promotion Course Using Gagne’s Model of Instructional Design on Nursing Students: Quantitative Research and Development Study
Source: JMIR Nurs. 2020 Nov 13;3(1):e19780. doi: 10.2196/19780 (PMC8279452; doi:10.2196/19780)
Supplement: Multimedia Appendix 2 [file nursing_v3i1e19780_app2.docx]

Tabel 2 Rerata skor SUS per butir pernyataan

| No | **Item Pernyataan** | Rerata |
| --- | --- | --- |
| 1 | Saya berpikir akan menggunakan system ini lagi | 3,03 |
| 2 | Saya merasa system ini rumit untuk digunakan | 2,95 |
| 3 | Saya merasa system ini mudah untuk digunakan | 3,05 |
| 4 | Saya membutuhkan bantuan dari orang lain atau teknisi dalam menggunakan sistem ini. | 2,56 |
| 5 | Saya merasa fitur – fitur system ini berjalan dengan semestinya. | 3,15 |
| 6 | Saya merasa ada banyak hal yang tidak konsisten (tidak serasi) pada system ini | 2,87 |
| 7 | Saya merasa orang lain akan memahami cara menggunakan system ini dengan cepat | 3,05 |
| 8 | Saya merasa system ini membingungkan | 3,03 |
| 9 | Saya merasa tidak ada hambatan dalam menggunakan system ini | 3,03 |
| 10 | Saya perlu membiasakan diri terlebih dahulu sebelum menggunakan system ini | 2,18 |

Sumber: Data Primer, 2019
